# Balancing Challenges and Opportunities When Evaluating Remote Rehabilitation for Sarcopenia in Older Adults

**DOI:** 10.2196/71845

**Published:** 2025-04-01

**Authors:** Hao Zhang, Xiangjie Liu

**Affiliations:** 1 Department of Geriatrics Liyuan Hospital, Tongji Medical College Huazhong University of Science and Technology Wuhan China

**Keywords:** telerehabilitation, elderly, sarcopenia, resistance exercise, rehabilitation, gerontology, aging, randomized controlled trial, rehabilitation training, body composition, strength, balance, cardiorespiratory endurance, self-care, physical therapy

Dear Editor:

We are writing to share our thoughts on the article “A 4-Week Mobile App–Based Telerehabilitation Program vs Conventional In-Person Rehabilitation in Older Adults With Sarcopenia: Randomized Controlled Trial” [1] published in the *Journal of Medical Internet Research*. This research presents a significant exploration into the rehabilitation of older adults with sarcopenia, comparing a mobile app–based telerehabilitation approach with traditional in-person rehabilitation. However, several elements of the study are worthy of in-depth discussion.

First, while the rehabilitation guidance provided by the mobile app included video and text instructions, the absence of real-time supervision may lead to variations in the older adult participants’ understanding and execution of the exercises. This lack of standardization in implementation across individuals could potentially influence the study’s outcomes [2]. Although the remote rehabilitation approach is innovative, concerns about the standardization of interventions remain. Furthermore, the inclusion criteria required participants to be capable of operating smartphones and possess basic literacy skills, but the study did not report the average age of the participants. This exclusion likely eliminated a significant portion of older adult patients with sarcopenia, potentially resulting in selection bias and limiting the generalizability of the findings to all older individuals with this condition. Future efforts could focus on developing more standardized and automated methods to ensure the accurate execution of exercises in remote rehabilitation programs. For example, incorporating artificial intelligence–based, motion-tracking technology into mobile apps could provide participants with real-time feedback.

Second, the universality of remote rehabilitation interventions warrants further consideration. Conducted within a specific hospital setting, the study’s effectiveness may be significantly diminished in regions with limited technological infrastructure or among patient groups with varying levels of smartphone and internet access [3]. Moreover, detailed information on participants’ comorbidities and medication regimens is crucial. The presence of chronic diseases and the use of various medications can significantly influence muscle function, metabolism, and responses to rehabilitation training. Neglecting these factors may undermine the accuracy of evaluating the effectiveness of the intervention.

Third, although participants were instructed to maintain consistent dietary habits, other factors such as daily activity levels and sleep quality, which also affect muscle condition and rehabilitation outcomes, were not strictly monitored or controlled. This lack of oversight may have introduced variables that interfere with the study’s results. Moreover, the impact of rehabilitation training on patients’ mental health (eg, improvements in depression and anxiety) and social functioning (eg, changes in participation in social activities) is equally significant. Future research could incorporate comprehensive nutritional assessments and interventions to better understand the combined effects of nutrition and exercise on sarcopenia.

As sarcopenia increasingly threatens the health of the older adult population, the findings of this study offer innovative perspectives on rehabilitation treatment. Future research should enhance the standardization and scalability of interventions, as well as improve data collection and evaluation systems. These efforts will not only provide deeper insights into the long-term effects of rehabilitation on older adults with sarcopenia but also drive rehabilitation medicine toward greater precision and efficiency.
